# Investigation of Pre- and Post-Weaning Mortalities in Rabbits Bred in Egypt, with Reference to Parasitic and Bacterial Causes

**DOI:** 10.3390/ani10030537

**Published:** 2020-03-24

**Authors:** Saeed El-Ashram, Shawky M. Aboelhadid, El-Sayed M. Abdel-Kafy, Shymaa A. Hashem, Lilian N. Mahrous, Eman M. Farghly, Asmaa A. Kamel

**Affiliations:** 1 College of Life Science and Engineering, Foshan University, 18 Jiangwan street, Foshan 528231, China; 2 Faculty of Science, Kafrelsheikh University, Kafr el-Sheikh, 33516, Egypt; 3 Parasitology Department, Faculty of Veterinary Medicine, Beni Suef University, Beni-Suef 62511, Egypt; dr.lilian_nagy@yahoo.com (L.N.M.); drasmaaalaa@yahoo.com (A.A.K.); 4 Animal Production Research Institute, Agricultural Research Center, Dokki, Giza 12651, Egypt; sayedabdkaffy@yahoo.com (E.-S.M.A.-K.); shaimaaahmedparavet@yahoo.com (S.A.H.); hawk18922@gmail.com (E.M.F.)

**Keywords:** pre- and postweaning, rabbits, mortality, *Eimeria*, *Salmonella*, *E. coli*, management

## Abstract

**Simple Summary:**

Pre- and postweaning stages are critical in the management of rabbits due to the increased risk of mortalities. Mortality rates during pre- and postweaning periods were 67.10% and 31.90%, respectively. The preweaning mortality was mainly due to causes related to does (95.23%) and infectious agents, including *Escherichia coli*, and *Salmonella* (4.77%). The postweaning mortality was mainly referred to managemental factors and infectious causes, including *Eimeria* species, *E. coli*, and *Salmonella*.

**Abstract:**

This study was conducted to investigate the causes of mortality in young rabbits. A total of 110 V-Line breed female rabbits aged 5 m were used in this study. Rabbit kits were examined daily in pre- and postweaning stages to detect clinical disorders that caused death. The postmortem examination was carried out on dead kits. Furthermore, rabbits were examined for the probable bacteriological and parasitological causes of death. Fecal samples were collected from each dead kit and examined by standard microbiological procedures for bacterial pathogens and macroscopically and microscopically for the presence of endo- and ectoparasites. Throughout two breeding seasons, 2238 newborns were obtained, of which 1736 died, accounting for a 77.57% mortality rate. During preweaning (1st month of age) and postweaning (up to 3 months of age), 1501 (67.10%) and 235 (31.90%) deaths were recorded, respectively. A postweaning fecal examination revealed that 198 out of 229 (86.50%) were diarrheic rabbits due to *Eimeria* infection. *Cittotaenia* spp. eggs were detected in 4.37% of fecal samples, and mites (*Sarcoptis scabiei*) were present in 6.55%. *E. coli* was detected in 100% of examined animals during pre- and postweaning periods; however, *Salmonella* spp. were 97.22% and 43.67, respectively. Managemental risk factors were the main causes in preweaning mortality, including insufficient milk supply (37.37%), cannibalism (26.38%), mange infestation of a rabbit doe (22.20%), mastitis (4.30%), lack of doe care (5.00%), bronchopneumonia (2.13%), and enteritis (1.80%). However, risk factors in postweaning mortality included sudden death with general septicemia (13.80%), enteritis (9.63%), bronchopneumonia (5.43%), mange infestation (2.04%), and malnutrition (0.81%). In conclusion, the etiology of preweaning mortality in kits was related mainly to the doe, especially managemental risk factors. However, a combination of multiple pathogenic agents (parasites and bacteria) and managemental factors was reported in the postweaning stage. Careful attention must be paid to avoid these causes.

## 1. Introduction

Rabbits play a significant role in solving the problem of protein shortage in many parts of the world due to their high fertility, prolificity, and rapid growth. However, pre- and postweaning mortalities could seriously damage the domestic rabbit industry, which affects lower-income families [1]. Weaning, which is a crucial period in the productive cycle of rabbits, has been associated with increased stress and susceptibility to diseases [2]. Moreover, establishing causes of pre- and postweaning mortalities remain difficult because multiple causes included either pathogenic agents (parasites, bacteria or viruses) or managemental factors in addition to adverse climatic conditions [3,4]. While 50% of mortality causes in rabbits are still unknown, parasites (*Eimeria* spp.) and bacteria (enteropathogenic *Escherichia coli*) remain the primary cause of nest mortality [5]. Coccidiosis is responsible for high losses in rabbit production and is considered a risk factor for intestinal disorders in the early postweaning period [6] and for liver condemnations as a result of hepatic coccidiosis [7]. Otherwise, sarcoptic mange infestation is one of the most common and major problems in the rabbit industry [8,9]. Sarcoptic mange mites are highly contagious and spread from rabbit to rabbit by direct skin contact or indirectly through fomites [9]. Severe sarcoptic mange infestations especially in young or debilitated rabbits cause high mortality [10]. The causes, such as infanticide, insufficient milk, birthing outside the nest box, mastitis, abscesses, genital infections, and death of dam may result in nest mortality. In addition, genetic and environmental factors may affect growth variation [4,11]. The aim of this study was to investigate the main parasitic, bacterial, and managemental causes of mortality in young rabbits during pre- and postweaning stages.

## 2. Materials and Methods 

### 2.1. Study Location and Ethics

This study was conducted in Sids station for animal breeding in Beni-Suef, Egypt, which belongs to the Animal Production Researches Institute (APRI), Agriculture Research Center (ARC), Ministry of Agriculture. The ethical rules for animal regulations were followed and approved by Beni-Suef University committee in January 2017 (BSU-19-2017). 

### 2.2. Period of the Study

This study was carried out continuously for two consecutive breeding seasons (2017–2019). Mortality rates were recorded for each season to assess the effect of infectious agents and managemental factors on the newborn survival rate. No births were recorded during summer as there was no breeding during the hot season.

### 2.3. Rabbit Husbandry and Management 

A total of 110 V-Line breed adult female rabbits (first-time mothers) aged 5 m was each kept in wire mesh cages (1 rabbit/cage), with natural ventilation, lighting, and ambient temperatures. Animals were fed ad libitum on commercial rabbit pelleted diet (not contain anticoccidial agents) in galvanized steel feeding hoppers and were provided water by automatic nipple drinkers. Each doe was identified by a numbered ear tag. About 3 d before birth, a nest box containing hay-straw bedding was placed in each cage. Does and their newborn kits were daily observed after birth, mainly for 3 d.

### 2.4. Recording of Production Data

A record sheet was prepared for each doe and kit on which to record all the detailed information, including doe number; date of birth; number of delivered; total litter size/birth; and maternal and newborn conditions, such as milk yield, disease conditions, mange infestation, abscessation of mammary glands, respiratory and digestive infections, and cannibalism signs.

### 2.5. Reporting Causes of Pre-and Postweaning Mortalities

#### 2.5.1. Preweaning Stage

About 2238 kits were born in two consecutive breeding seasons. Does were daily examined for maternal behavior and a swollen and whitish belly as a sign of initial milk feeding, while kits were observed for an increase in body mass as an indicator to normal growth. Dead kits were daily collected for postmortem (PM) examination. Furthermore, affected organs, including liver, lungs, trachea, kidneys, and alimentary tract, in addition to cecal content were collected from dead kits for microbiological and parasitological examinations to identify the causative agents that may be associated with mortality. The does were inspected for any disorders that affected newborn.

#### 2.5.2. Postweaning Stage

A total of 737 rabbits weaned at 35 days of age were separated from their does and transferred to new cages after inspection for postweaning disorders. Weaned rabbits were daily examined for clinical disorders associated with mortality in rabbits, including bloating, diarrhea, nasal discharges, and mange infestation. Dead rabbits were collected for postmortem (PM) examination, and organs with macroscopic lesions were sampled and sent to the laboratory for bacterial and parasitological examination. 

### 2.6. Investigation of Pathogenic Causes of Pre-and Postweaning Mortalities in Rabbits

#### 2.6.1. Parasitological Examination of Fecal Samples

A total of 301 fecal samples were obtained from young rabbits suffering from diarrhea. The samples represented 72 kits in the preweaning stage and 229 rabbits in the postweaning stage. Fecal samples were placed in separate labeled clean plastic cups to prevent potential cross contamination between samples and were transported on ice to the laboratory. Fecal samples were examined macroscopically and microscopically (Labomed Inc., New York, NY, USA) for their physical appearance and parasites, respectively [12]. A direct fecal smear was prepared from each fecal sample and examined for protozoal vegetative forms, cysts, and helminth eggs. The salt flotation technique was used to detect *Eimeria* oocysts [13]. The obtained *Eimeria* oocysts from fecal samples were separately stored into 2.5% potassium dichromate solution at 27 °C and 60%–80% humidity for 7 d [14]. Sporulated oocysts were collected after centrifugation (Centurion, Hoddesdon, UK), washed using distilled water, and identified under a microscope.

#### 2.6.2. Mange Examination 

Scabs were obtained from the edges of skin lesions by a scalpel from 15 postweaning rabbits suffering from mange clinical signs, were placed into plastic tubes, and were transferred to the laboratory. Scabs were placed in Petri dishes, incubated at 35 °C for 30 min in a biochemical oxygen demand (BOD) incubator (Velp, Usmate Velate MB, Italy), and examined microscopically for the presence of mites [15]. 

#### 2.6.3. Bacteriological Examination

A total of 301 young rabbits (72 from preweaning and 229 from postweaning) were found dead and examined for clinical signs. Affected organs, including liver, lungs, trachea, kidneys, and alimentary tract, that showed congestion, enlargement, and inflammation were collected in sterile plastic bags and kept in the ice box for examination. Tissue samples were screened for the presence of *Staphylococcus*, *Salmonella*, and *E. coli.* Bacterial colonies were identified according to previously described standard procedures [16,17,18,19]. Polymerase chain reaction (PCR) experiments were carried out following procedures previously published to confirm the identification of the isolates (Labnet Gradient PCR, Edison, NJ, USA) [20].

### 2.7. Statistical Analysis

All data were coded, entered, and analyzed using the statistical package SPSS version 22 (IBM Corp. Released 2013. IBM SPSS Statistics for Windows, Version 22.0., Armonk, NY, USA). Quantitative value data were summarized and described as frequencies and percentages. The relations between data grouped were tested by the Chi-square test for quantitative variables, and *p*-values were calculated if less than or equal to 0.05 (*p* ≤ 0.05) were considered significant.

## 3. Results

### 3.1. Mortality Rates 

**Figure 1:**
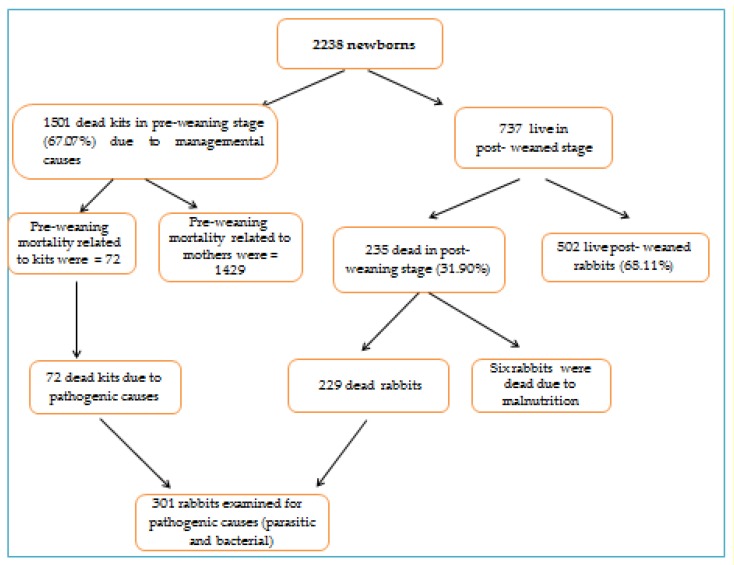
Flow chart showing the causes of newborn deaths in pre-and postweaning stages.

In the two consecutive breeding seasons (the combined effects of two consecutive breeding seasons), 2017–2018 and 2018–2019, a total of 2238 kits were born from 110 does. The total kit mortality rate was 77.57% (1736/2238), including preweaning (67.07%, 1501/2238) and postweaning (31.89%, 235/737) periods (Figure 1).

### 3.2. Managemental Causes of Preweaning Mortality 

The preweaning mortality rate was 67% over the two consecutive seasons. Causes of mortality related to does (1429/1501) were insufficient milk supply within the first few days after birth (37.37%), cannibalism (26.38%), mange infestation of does (22.20%), and mastitis (4.30%). Moreover, some does neglected their kits, which led to death of the whole litter (5.00%). Mortality rates related to the kits (72/1501) were bronchopneumonia (2.13%) and enteritis (1.80%). Sudden death without obvious causes was recorded in few cases 0.90% (Table 1). There was a significant difference (*p* < 0.05) between different managemental causes of preweaning mortality.

### 3.3. Pathogenic Causes of Preweaning Mortality in Rabbits

#### Bacteriological Causes of Preweaning Mortality

**Figure 2:**
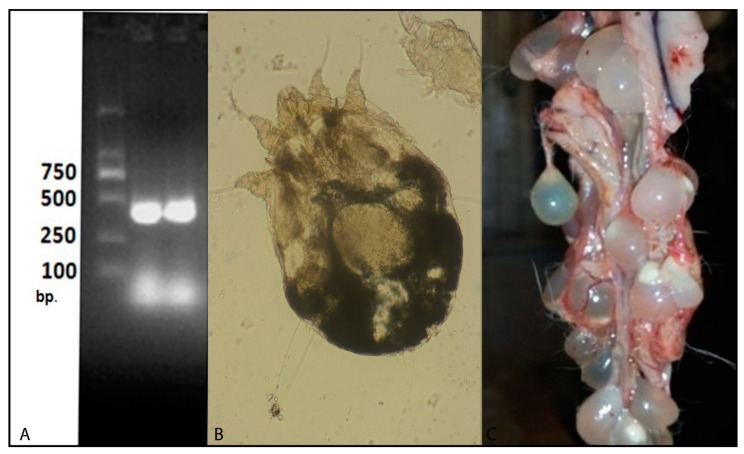
(**A**) *Cysticercus pisiformis* from the peritoneal cavity of rabbits, (**B**) *Sarcoptes scabiei* from the rabbit skin, and (**C**) analysis of *Salmonella* pure culture with the primer set: ST11-ST15. Lane 1 and Lane 2: *Salmonella.*

Examination of 72 dead kits in the preweaning stage revealed the presence of *E. coli* in all the 72 animals. Moreover, *Salmonella* was found in 97.22% of preweaned rabbits. PCR assay confirmed the identification of *Salmonella* species (Figure 2A). No parasitic causes were determined in the preweaning stage.

### 3.4. Seasonal Prevalence of Preweaning Mortality

Prevalence of preweaning mortality was highest during winter (36.5%) followed by autumn (32.6%) and spring (30.9%). In winter, the mortality as a result of insufficient milk supply, cannibalism, bronchopneumonia, and sudden death were recorded at the highest rates comparing to other seasons (Table 2). Meanwhile, mastitis and enteritis were the highest prevalence in spring. However, autumn showed the highest rate of mortality due to lack of mothering care (Table 3). The seasonal effect on preweaning mortality showed a significant difference (*p* < 0.05) between different seasons.

### 3.5. Managemental Causes of Postweaning Mortality

The prevalence of postweaning mortality rate was 31.90% (235/737) (Figure 1). Sudden death with symptoms of general septicemia in the muscles and internal organs was 13.80% followed by enteritis (9.63%), while the prevalence of bronchopneumonia was 5.43%. Moreover, the prevalence of mange mites was 2.04% and of malnutrition was 0.81% (Table 3). There was significant difference (*p* < 0.05) between different causes of postweaning mortality.

### 3.6. Pathogenic Causes of Postweaning Mortality in Rabbits

#### 3.6.1. Parasitological Findings of Postweaning Mortality

**Figure 3:**
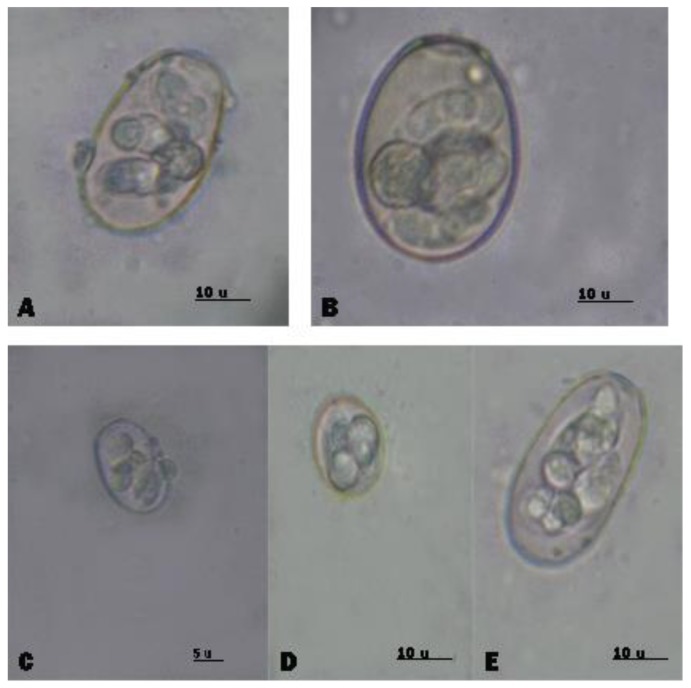
Sporulated oocysts of different species of *Eimeria* isolated from rabbits: (**A**) *E. media*, (**B**) *E. intestinalis*, (**C**) *E. magna*, (**D**) *E. exigua*, and (**E**) *E. coecicola*.

Examination of 229 fecal samples from diarrheic rabbits revealed that 86.50% (198/229) of rabbits were positive for *Eimeria* spp. Microscopic identification of eimerian oocysts revealed the presence of seven *Eimeria* species including (*E. media*, *E. perforans*, *E. intestinalis*, *E. magna*, *E. coecicola*, *E. exigua*, and *E. flavescens*). *E. media* was the most prevalent species (Figure 3). Furthermore, *Cittotaenia spp*. eggs were detected in 4.37% of fecal samples (Table 4). Mites (*Sarcoptes scabiei*) were detected in 6.55% (15/229 postweaning rabbits) (Table 4, Figure 2B). There were significant differences (*p* < 0.05) between the frequencies of different parasitic causes in the postweaning period.

#### 3.6.2. Bacteriological Causes of Postweaning Mortality

*E. coli* was found in all 229 animals. Moreover, *Salmonella* was found in 43.67% of postweaning animals (Table 4). Significant differences (*p* < 0.05) were found between the frequencies of different bacterial causes of postweaning mortality.

### 3.7. PostMortem Lesions of Postweaning Mortality

All necropsied rabbits carried *Eimeria* spp., pointing to coccidiosis as a probable cause of the death. Necropsied carcasses showed tinged blood and mucoid content in different parts of the intestinal tract with severe congestion and swelling. Furthermore, *Taenia pisiformis* cysts were attached to the peritoneal membrane in 4.37% (10/229) of dead rabbits (Figure 2C).

### 3.8. Seasonal Prevalence of Postweaning Mortality 

In the postweaning stage, the highest mortality rate was recorded in spring (48.9%) followed by winter (37.9%) while the lowest mortality rate was occurred in autumn (13.2%). No births were recorded in summer as breeding was stopped to avoid heat stress (Table 5). The seasonal effect on postweaning mortality showed a significant difference (*p* < 0.05) between different seasons.

## 4. Discussion 

Data on causes and rates of mortality are crucial for determining profitability and net income in commercial rabbit farming. However, studies on causes and rates of mortality on rabbit farms during pre- and postweaning stages are limited. This study investigated causes of pre- and postweaning mortality in 110 does during two consecutive breeding seasons. The overall mortality during preweaning was 67.07%. Similar findings were obtained by Ahmed et al. and Tameem et al. [11,21], who recorded 49.074% to 62.13% mortality rate in Egypt. However, the preweaning mortality rate in this study was higher than that recorded by Karu et al. [22], who observed that the preweaning mortality varies from 2.34% to 15.23% in New Zealand white kits. This variation could be attributed to the different causes of mortality affecting young rabbits including parasitic, bacterial, or managemental. The deaths during weaning in rabbits were very high due to many diseases affecting young kits at pre- and postweaning ages [23]. Interestingly, mother does have a major role in the mortality rate (95.23%) during the preweaning stage. Concerning the managemental causes related to mothers led preweaning mortality, the main cause of mortality was insufficient milk supply, which was associated with 37.37% of mortality. Mortality due to inadequate milk supply was more prominent in does that had large litter sizes which resulted in greater competition for doe teats during suckling and higher consumption of milk for fitter kits, which did not allow smaller and weaker kits to consume enough milk with resultant starvation and death. These findings are in line with those recorded by [24,25,26,27,28,29,30]. Prevalence of preweaning mortality due to insufficient milk supply and large litter size was the highest in winter (45.28%) while the lowest was in autumn (26.6%). Similar results were obtained by [31,32]. The prevalence of preweaning mortality because of cannibalism was 26.38%. This finding agrees with [33,34], who suggested that does tend to eat their young kits due to insufficient drinking water around parturition time. The prevalence of preweaning mortality due to mite infestation in mothers was 22.20%. Moreover, sarcoptic manage contributed to high mortality because does became clinically aggressive due to severe irritation and restlessness, which were made worse with low feed intake resulting in inappetence and loss of body weight with poor growth for young rabbits. Similar findings were reported by several studies [35,36,37,38]. *Sarcoptes scabiei* was detected in all mite-infected rabbits in the current study, and the highest prevalence of infestation was in autumn (63.96%) while the lowest was in winter (14.41%), which is consistent with previous findings [23,39]. Warm conditions and the high humidity may have favored rapid mite reproduction, spread, and infestation among rabbits [9]. Furthermore, mastitis was observed in 4.30% of does. Mastitis interferes with suckling and may lead to complete breast dryness and milk cessation. These findings are supported by [34,40]. The highest prevalence of mastitis was recorded in late spring (71.9%) while, in winter, was (28.1%) consistent with results previously reported by [41]. Concerning the pathogenic causes of preweaning mortality, *E.coli* and *Salmonella* were identified as bacterial causes of mortality with prevalence of 100% and 97.22% of preweaned rabbits. Similar findings were reported by [42]. It is worthy to mention that no parasitic causes were determined in preweaning stage. Our findings revealed that the highest prevalence of pre-weaning mortality was recorded in winter (36.5%) due to cold or lower temperatures, inadequate warming, and insufficient heat insulation, which led in decreased feed intake, reduced vitality, and poor body conditions. The lowest prevalence was recorded in spring (30.9%). However, preweaning mortality increased with the increase of ambient temperature that could be attributed to the heat stress and reduction of dam’s milk [40]. 

The prevalence of postweaning mortality was 31.90% (235/737); these results revealed that mortality decreased with growing age in accordance with previous studies [1,11,43]. Also, most of mortality causes were related to pathogenic agents. Differences in pre- and postweaning mortality may be attributed to several factors, including pathogenic, genetic, environmental and managemental conditions, and the maternal ability of mothers does as suggested by Rashwan [34]. Regarding the parasitic causes of mortality, oocysts of coccidia were a prominent cause of young rabbit mortality in this investigation. Different species of *Eimeria*,, including *E. media*, *E. perforans*, *E. intestinalis*, *E. magna*, *E. coecicola*, *E. exigua*, and *E. flavescens*, were detected in line with previous studies [6,7,44] which revealed that rabbit coccidiosis was an important cause of mortality within the first three to four days after birth in rabbits and that the main source of *Eimeria* spp. for young rabbits was *Eimeria* oocysts shed by adult rabbits. Sarcoptic mange (*Sarcoptes scabiei*) was also an important parasitic cause of mortality, which is in agreement with previous studies showing that sarcoptic mange was a highly contagious infectious disease, rapidly spreading parasitic disease in Egypt, and next to coccidiosis in the economic importance [9,37]. Furthermore, *E. coli* and *Salmonella* spp. were identified as probable bacterial causes of mortality as they were detected in 100% and 43.67% of rabbits, respectively. Similar findings were reported by Eid [42]. Necropsied rabbits revealed a number of lesions including enteritis 9.63% and bronchopneumonia (5.43%), which may have contributed to mortality. Pneumonia and enteritis may have been induced by parasitic and bacterial organisms. Both pneumonia and enteritis are major problems causing high mortalities in rabbitries [45]. Seasonal variations and climatic conditions play a vital role in rabbit survival due to the direct effect of ambient temperature on does and the young. The highest prevalence of postweaning mortality (48.9%) was recorded in late spring with the gradual rise in ambient temperature, whereas the lowest prevalence was in autumn (13.2%). Similar results were obtained previously in other studies [45,46,47], which reported that high ambient temperatures reduce the feed intake of lactating does from 25% to 50% leading to reduction energy and deterioration of doe vitality, body condition, and productivity. 

## 5. Conclusions

In conclusion, doe and kit health can be achieved by reducing preweaning mortality through adjustment of the rabbitry’s climatic conditions, by improving the managemental conditions, and by avoiding food and water contamination. Nevertheless, the results were based on a limited sample size (the combined effects of two consecutive breeding seasons), and larger well-designed studies (5 consecutive breeding seasons) are expected to confirm these preliminary findings.

## Figures and Tables

**Table 1:** Managemental causes of preweaning mortality related to mothers and kits.

Causes of Kit’s Death	Mortality Related to Mothers	Mortality Related to Kits	SEM	*p* *
Insufficient milk supply	561(37.37%)	0	1.109	0.000
Infanticidal does (cannibalism)	396 (26.38%)	0		
Mange infested does	333 (22.20%)	0		
Mastitis	64 (4.30%)	0		
Lack of mothering	75 (5.00%)	0		
Bronchopneumonia in litters	0	32 (2.13%)		
Enteritis in litters with diarrhea	0	27 (1.80%)		
Sudden death in litters	0	13 (0.90%)		
Total	1429 (95.23%)	72 (4.83%)		

SEM: standard error of mean. * *p* < 0.05 is significant.

**Table 2:** Seasonal prevalence of preweaning mortality.

Causes of Kit’s Death.	Total No. of Dead Kits	Autumn	%	Winter	%	Spring	%	SEM	*p* *
Insufficient milk supply	561	149	26.6	254	45.28	158	28.16	0.751	0.000
Mange infested does	333	213	63.96	48	14.41	72	21.62		
Infanticidal does	396	47	11.87	201	50.76	148	37.37		
Mastitis in lactating females	64	-	0	18	28.1	46	71.9		
Lack of mothering	75	65	86.7	-	0	10	13.3		
Bronchopneumonia	32	5	15.6	15	46.9	12	37.5		
Enteritis	27	7	25.9	6	22.2	14	51.9		
Sudden death	13	3	23	6	46.15	4	30.8		
Total	1501	489	32.6	548	36.5	464	30.9		

SEM: Standard error of mean. * *p* < 0.05 is significant.

**Table 3:** Prevalence of postweaning mortality related to the cause of death.

Causes of Death	Number of Dead Kits/737	%	SEM	*p* *
Sudden death with Septicemia	103	13.80	0.725	0.000
Diarrhea (enteritis)	71	9.63		
Bronchopneumonia	40	5.43		
Mange infestation	15	2.04		
Malnutrition	6	0.81		
Total	235	31.90		

SEM: Standard error of mean. * *p* < 0.05 is significant.

**Table 4:** Prevalence of pathogenic causes in postweaning periods in rabbits.

Pathogenic Agents	Postweaning Mortality (*n* = 229)	Prevalence %	SEM	*p* *
*Eimeria* spp. Oocysts	198	86.50	1.26	0.000
*Cittotaenia* spp. Eggs	10	4.37		
*Sarcoptes scabiei*	15	6.55		
*E. coli*	229	100		
*Salmonella*	100	43.67		
*Eimeria* spp. + *E. coli*	198	86.50		
*Eimeria* spp. + *Salmonella*	100	43.67		

SEM: Standard error of mean. * *p* < 0.05 is significant.

**Table 5:** Seasonal prevalence of postweaning mortality.

Season	Postweaning Mortality	%	SEM	*p* *
Autumn	31	13.2	0.638	0.008
Winter	89	37.9		
Spring	115	48.9		
Total	235	31.90		

SEM: Standard error of mean. * *p* < 0.05 is significant.

## References

[1] Rashwan A.A., Ahmed S.A. Growing rabbit management, housing system, reduction of eating time and feeder space. Proceedings of the 6th World Rabbit Congress.

[2] Millar J.S. (2007). Nest mortality in small mammals. Ecoscience.

[3] Heppell S.S., Caswell H., Crowder L.B. (2000). Life histories and elasticity patterns: Perturbation analysis for species with minimal demographic data. Ecology.

[4] Oli M.K., Dobson F.S. (2003). The relative importance of life-history variables to population growth rate in mammals: Cole’s prediction revisited. Am. Nat..

[5] Licois D. Domestic rabbit enteropathogens. Proceedings of the 8th World Rabbit Congress.

[6] El-Ashram A.S., Aboelhadid S.M., Abdel-Kafy E.M., Hashem S.A., Mahrous L.N., Farghly E.M., Moawad U.K., Kamel A.A. (2019). Prophylactic and Therapeutic Efficacy of Prebiotic Supplementation against Intestinal Coccidiosis in Rabbits. Animals.

[7] Pakandl M. (2009). Coccidia of rabbit: A review. Folia Parasitol..

[8] Darzi M.M., Mir M.S., Shahardar R.A., Pandit B.A. (2007). Clinicopathological, histochemical and therapeutic studies on concurrent sarcoptic and notoedric acariosis in rabbits *(Orytolagus cuniculus)*. Vet. Orhiv..

[9] Aboelhadid S.M., Mahrous L.N., Hashem S.A., Abdel-Kafy E.M., Miller R.J. (2016). In vitro and in vivo effect of Citrus limon essential oil against sarcoptic mange in rabbits. Parasitol. Res..

[10] Bornstein S.M.T., Samuel W.H. (2001). Parasitic Diseases of Wild Mammals.

[11] Tameem Eldar A.A., Elamin K.M., Yousif I.A., Hassan H.E., Musa A.M. (2012). Growth and Mortality in Pre-and Post-Weaning Rearing Periods for Sudanese Local Rabbits. J. Anim. Prod. Adv..

[12] Soulsby E.J.L. (1986). Helminths, Arthropods and Protozoa of Domesticated Animals.

[13] Zajac A.M., Conboy G.A. (2006). Veterinary Clinical Parasitology.

[14] Huang G., Zhang S., Zhou C., Tang X., Li C., Wang C., Tang X., Suo J., Jia Y., El-Ashram S. (2018). Influence of *Eimeria falciformis* infection on Gut Microbiota and Metabolic Pathways in Mice. Infect. Immun..

[15] Fichi G., Flamini G., Zaralli L., Perrucci S. (2007). Efficacy of an essential oil of *Cinnamomum Zeylanicum* against *Psoroptes cunicul*. Phytomedicine.

[16] Holt J.G., Krieg N.R., Sneath P.H., Staley J.T., Williams S.T. (1994). Bergey’s Manual of Determinate Bacteriology.

[17] ISO 6888-2:1999 (1999). International Organization for Standardization. Microbiology of Food and Animal Feeding Stuffs—Horizontal Method for the Enumeration of Coagulase-Positive Staphylococci (Staphylococcus aureus and other species) e Part 2: Technique Using Rabbit Plasma Fibrinogen Agar Medium.

[18] ISO 6579:2002 (2002). International Organization for Standardization. Horizontal Method for the Detection of Salmonella spp. Microbiology of Food and Animal Feeding Stuffs.

[19] Lee M., Arp L., Swayne D.E. (1998). Colibacillosis. A Laboratory Manual for the Isolation and Identification of Avian Pathogens.

[20] Soumet C., Ermel G., Rose V., Rose N., Drouin P., Salvat G., Colin P. (1999). Identification by a multiplex PCR-based assay of *Salmonella typhimurium* and *Salmonella* enteritidis strains from environmental swabs of poultry houses. Lett. Appl. Microbiol..

[21] Ahmed S., Marai I.F.M. Milk yield and litter traits of rabbits as affected by breed, parity, number of nipples, and aminazine injection, under Egyptian sub-tropical conditions. Proceedings of the First International Conference for Animal Production and Health in Semi-Arid Area.

[22] Karu P., Muthusamy P., Gopi H., Balasubramanyam D., Babu M. (2014). Survivability in New Zealand White breed of rabbits under farming condition in Tamilnadu. Int. Sci. Environ. Technol..

[23] Mohammed H.A., Eid A.A.M., El-Bakrey R.M.M. (2013). A review of rabbit diseases in Egypt. Wartazoa.

[24] Drummond H., Vázquez E., Sánchez–Colón S., Martinez–Gómez M., Hudson R. (2000). Competition for milk in the domestic rabbit: Survivors benefit from littermate deaths. Ethology.

[25] Poigner J., Szendrộ Z., Lèvai A., Radnai I., Birớ-Nemeth E. (2000). Effect of birth weight and litter size at suckling age on reproductive performance in does as adults. World Rabbit Sci..

[26] Castellini C., Dal Bosco A., Mugnai C. (2003). Comparison of different reproduction protocol for rabbit does: Effect of rabbit production in tropical and sub-tropical agricultural system. J. Amin. Sci..

[27] Bautista A., Mendoza-Degante M., Coureaud G., Martinez-Gómez M., Hudson R. (2005). Scramble competition in newborn domestic rabbits for an unusually restricted milk supply. Animal Behav..

[28] Hudson R., Trillmich F. (2008). Sibling competition and cooperation in mammals: Challenges, developments and prospects. Behav. Ecol. Sociobiol..

[29] Gotz A.A., Wolf M., Stefanski V. (2008). Psychosocial maternal stress during pregnancy: Effects on reproduction for F0 and F1 generation laboratory rats. Physiol. Behav..

[30] Rodel H.G., Prager G., Stefanski V., von Holst D., Hudson R. (2008). Separating maternal and litter size effects on early postnatal growth in two species of altricial mammals. Physiol. Behav..

[31] Bhatt R.S., Sharma S.R., Singh U., Kumar D., Bhasin V. (2002). Effect of different season on the performance of grey giant rabbits under sub-temperate Himalayan conditions. Asian-Australian J. Anim. Sci..

[32] Kumar D., Singh U., Bhatt R.S., Risam K.S. (2005). Reproductive efficiency of female German Angola under Indian sub- temperate climatic conditions. World Rabbit Sci..

[33] Rodel H.G., Starkloff A., Bautista A., Friedrich A.C., von Holst D. (2008). Infanticide and maternal offspring defence in European rabbits under natural breeding conditions. Ethology.

[34] Rashwan A.A., Marai I.F.M. (2000). Mortality in young rabbits: A review. World Rabbit Sci..

[35] Rashed E.A.O. (1993). Survey on Parasitic Diseases of Rabbits. Master Thesis.

[36] Dagleish M.P., Ali Q., Powell R.K., Butz D., Woodford M.H. (2007). Fetal *Sarcoptes scabiei* infection of blue sheep *(Pseudoisnayaur*) in Pakistan. J. Wildl. Dis..

[37] Bonanno A., Mazza F., Di Grigoli A., Alicata M.L. Body condition score and related productive responses in rabbit does. Proceedings of the 9th World Rabbit Congress.

[38] Rodel H.G., Starkloff A., Seltmann M.W., Prager G., von Holst D. (2009). Causes and predictors of nest mortality in a European rabbit population. Mamm. Biol..

[39] EL-Zemity S.R., Rezk H.A., Zaitoon A.A. (2006). Acaricidal activity of some essential oils and their monoterpenoidal constituents against the parasitic Bee mites *Varroa destructor* (Acari: Varroidae). J. Appl. Sci. Res..

[40] Rosell J.M., de la Fuente L.F. (2009). Culling and mortality in breeding rabbits. Prev. Vet. Med..

[41] Sánchez J.P., de la Fuente L.F., Rosell J.M. (2012). Health and body condition of lactating females on rabbit farms. J. Anim. Sci..

[42] Eid A.A.M., Ibraheem O.K. (2006). Sudden death among rabbits in Sharkia Province, Egypt. Zagazig Vet. J..

[43] Peeters J.E. Recent advances in intestinal pathology of rabbits and further prespectives. Proceedings of the 4th World Rabbit Congress.

[44] Shahin A.M., Lebdah M.A., Ali G.R.M. (2011). *Escherichia coli* as an etiological agent of mucoid enteropathy in rabbits. Researcher.

[45] Habeeb A.A.M., Marai I.F.M., El-Maghawry A.M., Gad A.A. (1997). Growth rabbits as effected by salinity in drinking water under winter and hot summer conditions of Egypt. Egyptian J. Rabbit Sci..

[46] Shehata A.S., Sarhan M.A., El-Gendy K.M. (1998). Digestibility, thyroid function and growth performance of New Zealand White rabbits as affected by season of the year and age. Egyptian J. Rabbit Sci..

[47] Fortun-Lamothe L. (2006). Energy balance and reproductive performance in rabbit does. Anim. Reprod. Sci..

