# Erratum: Saeed, E.-A.; Shawky, M.A.; El-Sayed, M.A.-K.; Shymaa, A.H.; Lilian, N.M.; Eman, M.F.; Asmaa, A.K. Investigation of Pre- and Post-Weaning Mortalities in Rabbits Bred in Egypt, with Reference to Parasitic and Bacterial Causes. *Animals* 2020, *10*, 537

**DOI:** 10.3390/ani10040650

**Published:** 2020-04-10

**Authors:** Saeed El-Ashram, Shawky M. Aboelhadid, El-Sayed M. Abdel-Kafy, Shymaa A. Hashem, Lilian N. Mahrous, Eman M. Farghly, Asmaa A. Kamel

**Affiliations:** 1 College of Life Science and Engineering, Foshan University, 18 Jiangwan street, Foshan 528231, China; 2 Faculty of Science, Kafrelsheikh University, Kafr el-Sheikh 33516, Egypt; 3 Parasitology Department, Faculty of Veterinary Medicine, Beni Suef University, Beni-Suef 62511, Egypt; dr.lilian_nagy@yahoo.com (L.N.M.); drasmaaalaa@yahoo.com (A.A.K.); 4 Animal Production Research Institute, Agricultural Research Center, Dokki, Giza 12651, Egypt; sayedabdkaffy@yahoo.com (E.-S.M.A.-K.); shaimaaahmedparavet@yahoo.com (S.A.H.); hawk18922@gmail.com (E.M.F.)

The authors wish to make the following correction to their paper [1]:

The caption of Figure 2 was referring to a different Figure and has now been corrected (see the corrected version of Figure 2 below):

## Figures and Tables

**Figure 2:**
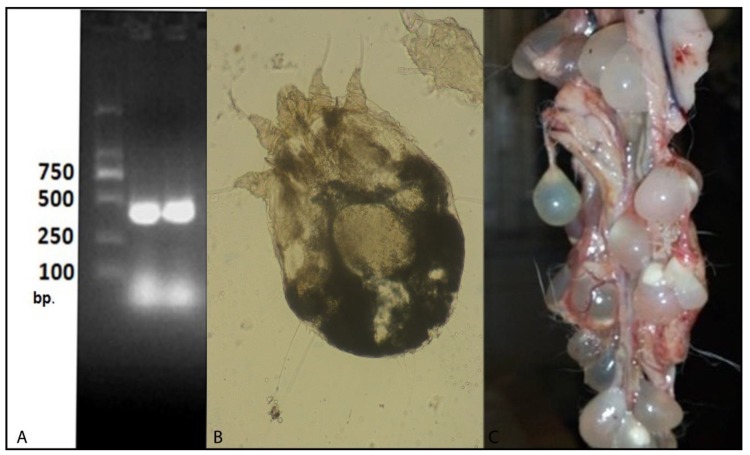
(**A**) Analysis of *Salmonella* pure culture with the primer set: ST11-ST15. Lane 1 and Lane 2: *Salmonella* (**B**) *Sarcoptes scabiei* from the rabbit skin, and (**C**) *Cysticercus pisiformis* from the peritoneal cavity of rabbits.
